# Supplementary material for: The importance of A-site cation chemistry in superionic halide solid electrolytes
Source: Nat Commun. 2024 Aug 29;15:7501. doi: 10.1038/s41467-024-51710-1 (PMC11362563; doi:10.1038/s41467-024-51710-1)
Supplement: Supplementary file 1 — Supplementary Information [file 41467_2024_51710_MOESM1_ESM.pdf]

Supplementary information

The Importance of A-site Cation Chemistry in Superionic Halide Solid Electrolytes

Kit Barker[1], Sarah L. McKinney[2,3,4], Raul Artal[5], Ricardo Jimenez[5], Nuria Tapia-Ruiz[2,4], Stephen J. Skinner[1], Ainara Aguadero[1,5] and *Ieuan D. Seymour[1,6]

[1]Department of Materials, Imperial College London, UK

[2]Department of Chemistry, Imperial College London, UK

[3]Department of Chemistry, Lancaster University, UK

[4]The Faraday Institution, Harwell Science and Innovation Campus, Didcot, UK

[5]Instituto de Ciencia de Materiales de Madrid, CSIC, Spain

[6] Advanced Centre for Energy and Sustainability (ACES), Department of Chemistry, University of Aberdeen, UK

*Corresponding author: ieuan.seymour08@imperial.ac.uk

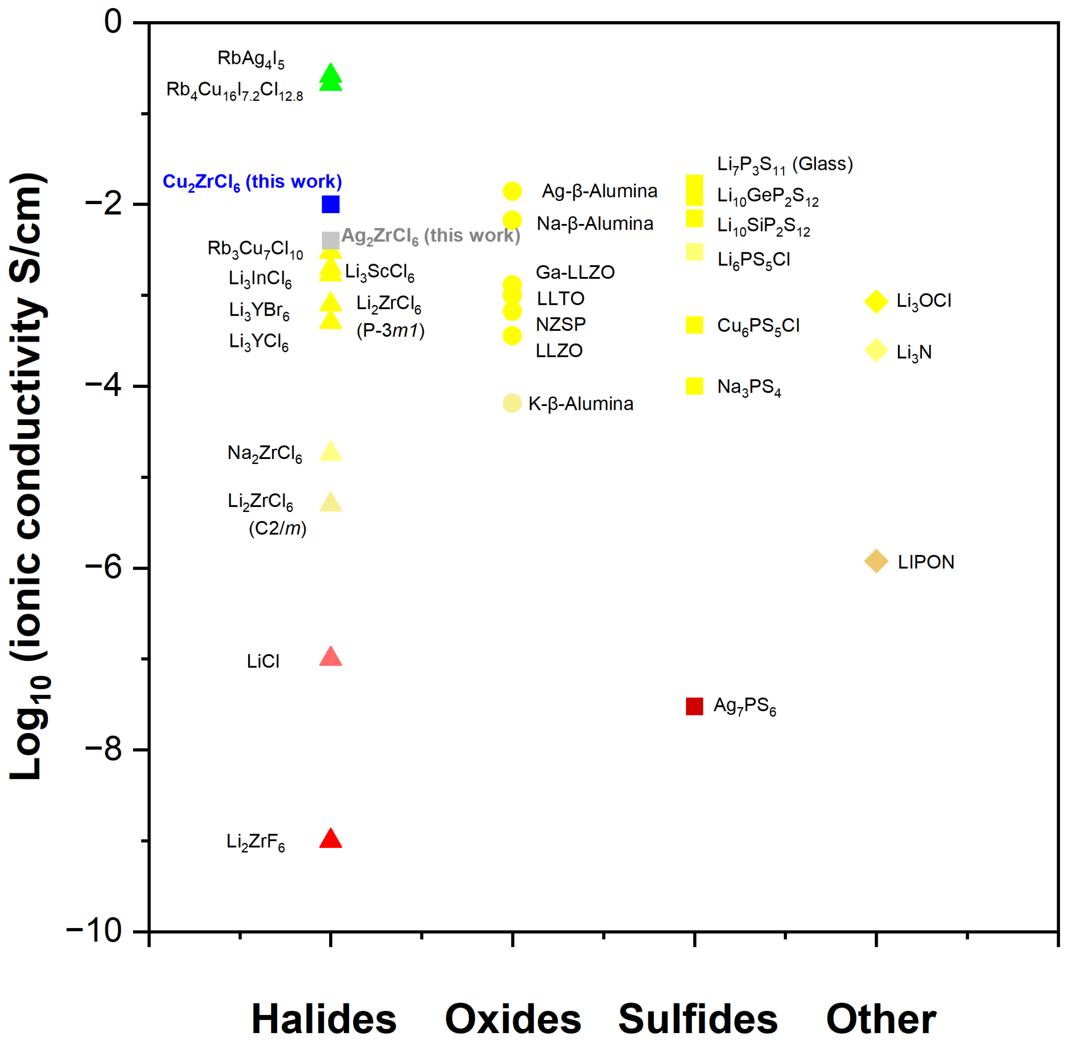

Supplementary Figure 1: Room temperature ionic conductivities of various ion conductors. See references [1-26] for further details.

Supplementary note 1: Structural considerations for ionic conduction in halides

In Na_2ZrCl_6, the more commonly reported $P\bar{3}m1$ structure reflects that of Li_3YCl_6[27]. The alternative $P2_1/n$ structure for Na_2ZrCl_6 contains prismatic Na^+ sites and octahedral Zr^{4+} sites. The reported ionic conductivity of Na_2ZrCl_6 is relatively low and depends heavily on the disorder in the system. Sebti *et al.*[27] showed that a slow cooled sample of Na_2ZrCl_6 displayed the lowest conductivity of their samples (6.6×10^{-8} S cm^{-1}) while a twice milled sample achieved ionic conductivities of up to 2.6 ×

10^{-5} S cm^{-1}, further supporting the idea that disorder is beneficial for fast ion transport in halide systems.

The main difference in Cu_2ZrCl_6 is that the Cu^+ ions were found to be located on tetrahedral sites rather than octahedral sites within the same HCP framework. Dattelbaum *et al.*[28]also found a fully ordered zirconium sublattice as opposed to partial occupancies across the octahedral sites. The copper ions form clusters of edge sharing tetrahedra around the vacant zirconium octahedral sites c = 0.5.

α-AgI, an advanced superionic conductor displays an ionic conductivity of around 1 S cm^{-1} above 146 °C[29] which is more than 4 orders of magnitude faster than the low temperature β-AgI phase. The average structure is considered to be where the silver ions are partially occupied across 1/6 of the face sharing tetrahedral sites[30]. The high conductivity in this system has been attributed to the high ratio of available sites to mobile charge carriers (Ag^+ ions) as well as the face sharing tetrahedra that provide a three dimensional transport network.

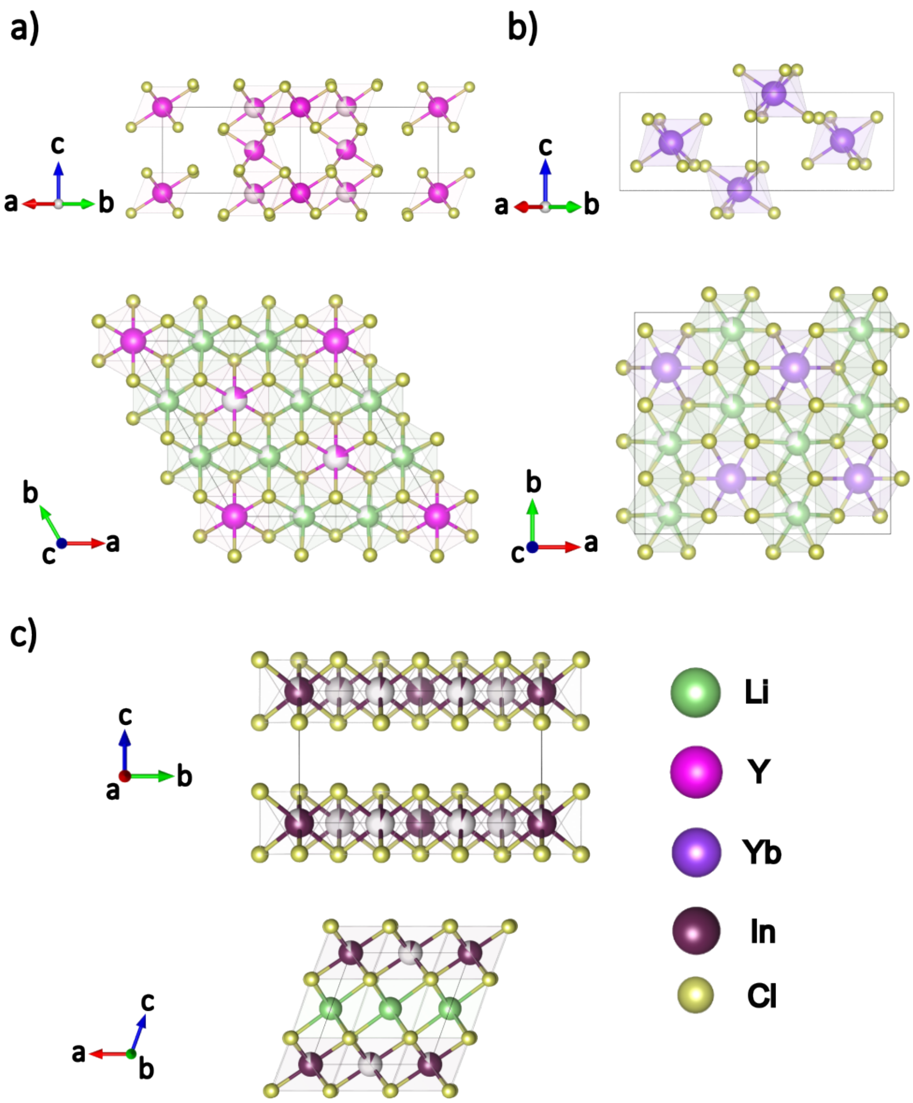

Supplementary Figure 2: Comparison of common halide solid electrolyte structures. (a) Structure of Li_3YCl_6 showing the large degree of disorder of cations within the $P\bar{3}m1$ space group. The Y in this structure is edge sharing with Li while face sharing with itself, Li in this structure is disordered over 2 sites. Li(1) positions are edge sharing with themselves but face sharing with Li(2) positions in the c-direction. The chloride sublattice is also HCP in this structure[31]. (b) The *Pnma* structure of Li_3YbCl_6. Yb is fully ordered and located on one site in a mixed Li, Yb layer. Li is disordered over two sites which are face sharing with each other. The chloride sublattice is HCP [32]. (c) Structure of Li_3InCl_6 showing the $InCl_6^{3-}$ octahedra and Li^+ positions. Li is ordered in octahedral sites of one layer while indium has partial disorder across the octahedral sites in a separate layer, both the $InCl_6^{3-}$ and $LiCl_6^{5-}$ are edge sharing and the Cl^- sublattice is CCP in nature[33].

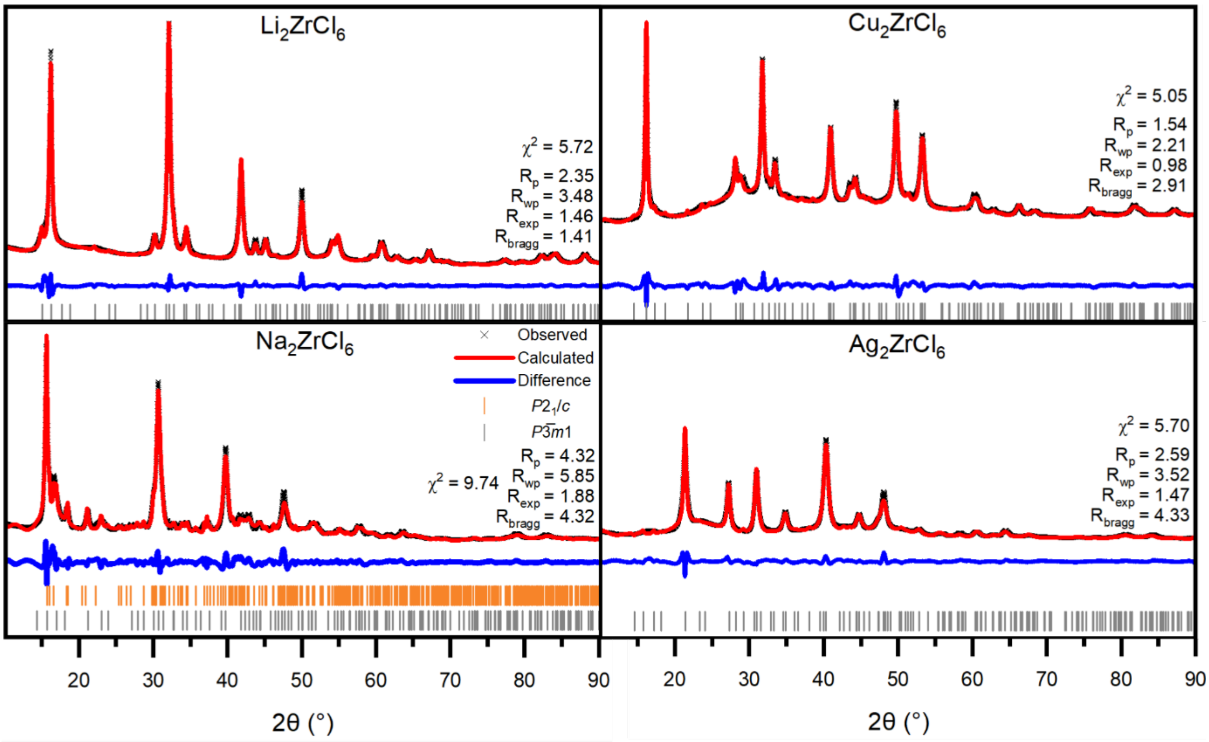

Supplementary Figure 3: Lab X-ray diffraction of mechanochemically synthesised *A*$_2$ZrCl$_6$ materials. Rietveld refinements of A_2ZrCl_6 using their respective fully disordered $P\bar{3}m1$ starting structures A secondary $P2_1/n$ phase was required to adequately fit the Na_2ZrCl_6 sample. The presence of this secondary phase led to a slightly higher χ^2 value (9.70) compared to the Li_2ZrCl_6, Ag_2ZrCl_6 and Cu_2ZrCl_6 refinements containing a single $P\bar{3}m1$ phase where $\chi^2 < 6$.The broadness of the peaks, associated with reduced crystallite sizes as a result of the harsh milling process, increases the difficulty of refining X-ray diffraction data. To avoid over fitting of broad peaks leading to non physical values, some isotropic thermal displacement parameters were fixed. The humps observed in the diffraction patterns of Cu_2ZrCl_6 and Ag_2ZrCl_6 may indicate a small amount of amorphous phase.

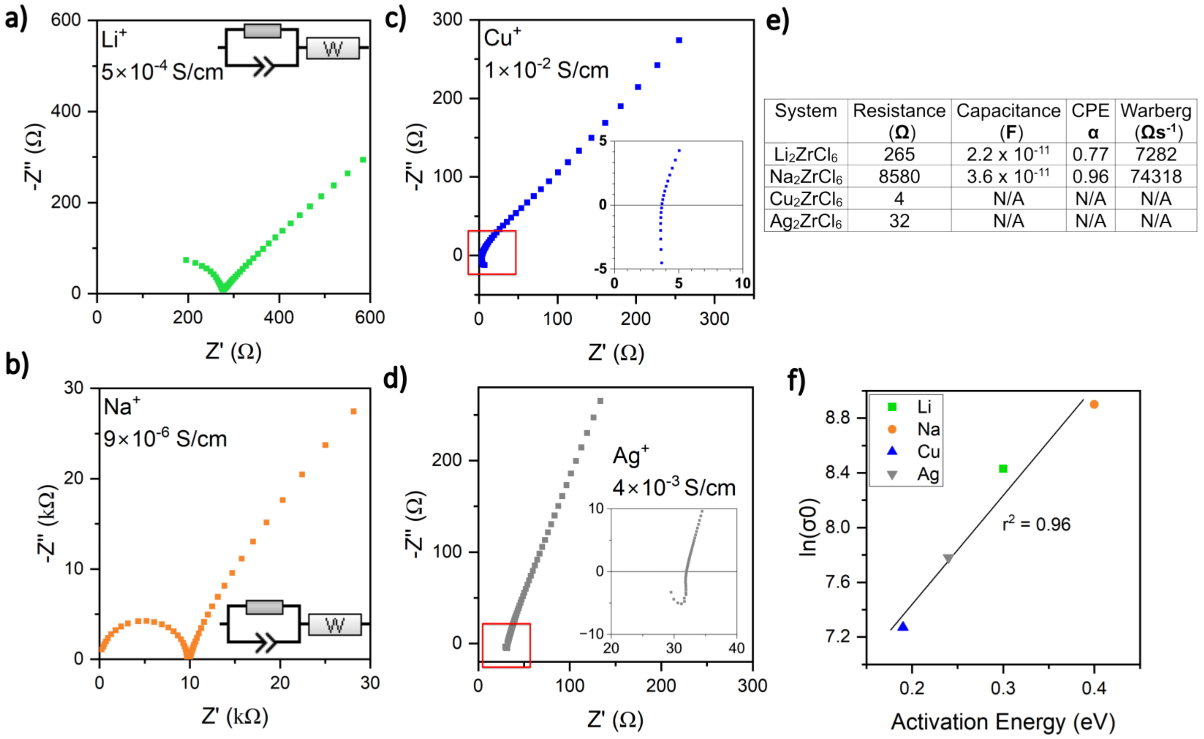

System	Resistance (**Ω**)	Capacitance (**F**)	CPE **α**	Warberg (**Ωs^{-1}**)
Li_2ZrCl_6	265	2.2×10^{-11}	0.77	7282
Na_2ZrCl_6	8580	3.6×10^{-11}	0.96	74318
Cu_2ZrCl_6	4	N/A	N/A	N/A
Ag_2ZrCl_6	32	N/A	N/A	N/A

Supplementary Figure 4: Ionic conductivity analysis of *A*$_2$ZrCl$_6$ materials. Nyquist plots for a) Li_2ZrCl_6, b) Na_2ZrCl_6, c) Cu_2Zr Cl_6 and d) Ag_2ZrCl_6. The equivalent circuit models consisting of a parallel resistor/constant phase element and a series Warburg element that were used to fit the resistance and capacitance of the Li_2ZrCl_6 and Na_2ZrCl_6 systems are shown in (a) and (b). The resistance for the Cu_2ZrCl_6 and Ag_2ZrCl_6 systems was taken from the x-intercept. All measurements taken at room temperature (25°C). e) Table containing the fitted data extracted via the equivalent circuit models. f) Plot of activation energy vs pre-exponential factor demonstrating a Meyer-Neldel conductivity relationship.

Supplementary note 2: EIS analysis

Semicircles are not observed for Cu_2ZrCl_6 or Ag_2ZrCl_6. The time constant of a conduction process is related to the resistance and capacitance of the system:

$$\tau = RC$$

The frequency *f* at which a process is observed in EIS is related to τ via the angular frequency ω:

$$\omega = 2\pi f$$

$$\omega = 1/\tau$$

As conductivity is inversely proportional to resistance, we can see that very large conductivities require increasingly higher frequencies (>7MHz in this case) to resolve the conduction process.

As shown in Supplementary Figure 4(e), the A_2ZrCl_6 system shows Meyer-Neldel behaviour in which a material with a higher activation energy exhibits higher ionic

conductivity than a material with a lower activation energy at high temperature, as the activation energy embedded within the pre-exponential factor becomes more dominant[35].

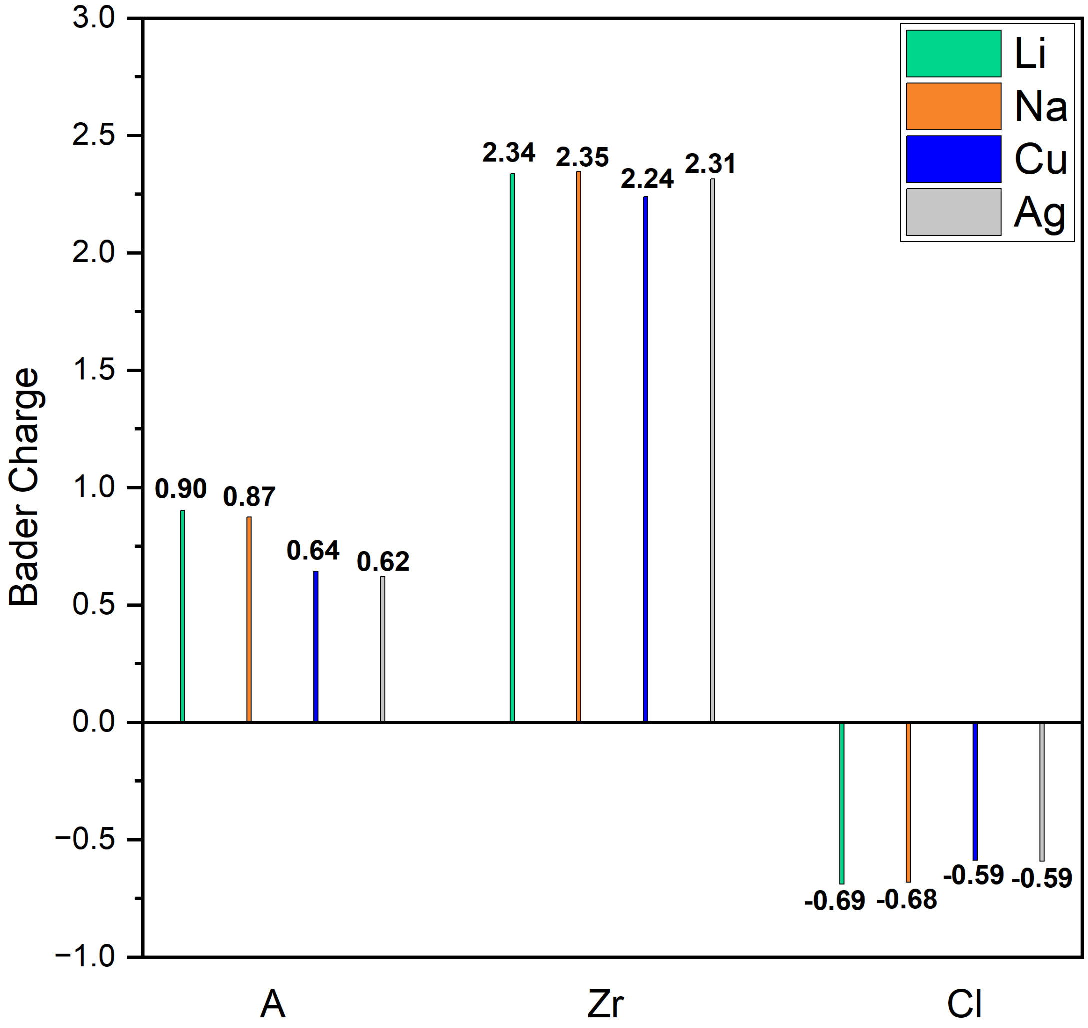

Supplementary Figure 5: Bader charge analysis. Mean Bader charges of the species in the ground states of A_2ZrCl_6.

Supplementary note 3: Bader charge analysis

Bader charge analysis is a way of determining the distribution of electronic charge within a molecule or solid by defining the contributions from individual atoms. This is achieved by finding where the electron density gradient is zero, representing the areas of bonding between atoms. These areas of electron density can be used to find the charge associated with each atom. These charges can be compared to that of the species formal charges to see the bonding character between species. Bader charge analysis was performed using the Bader charge analysis code.[36]

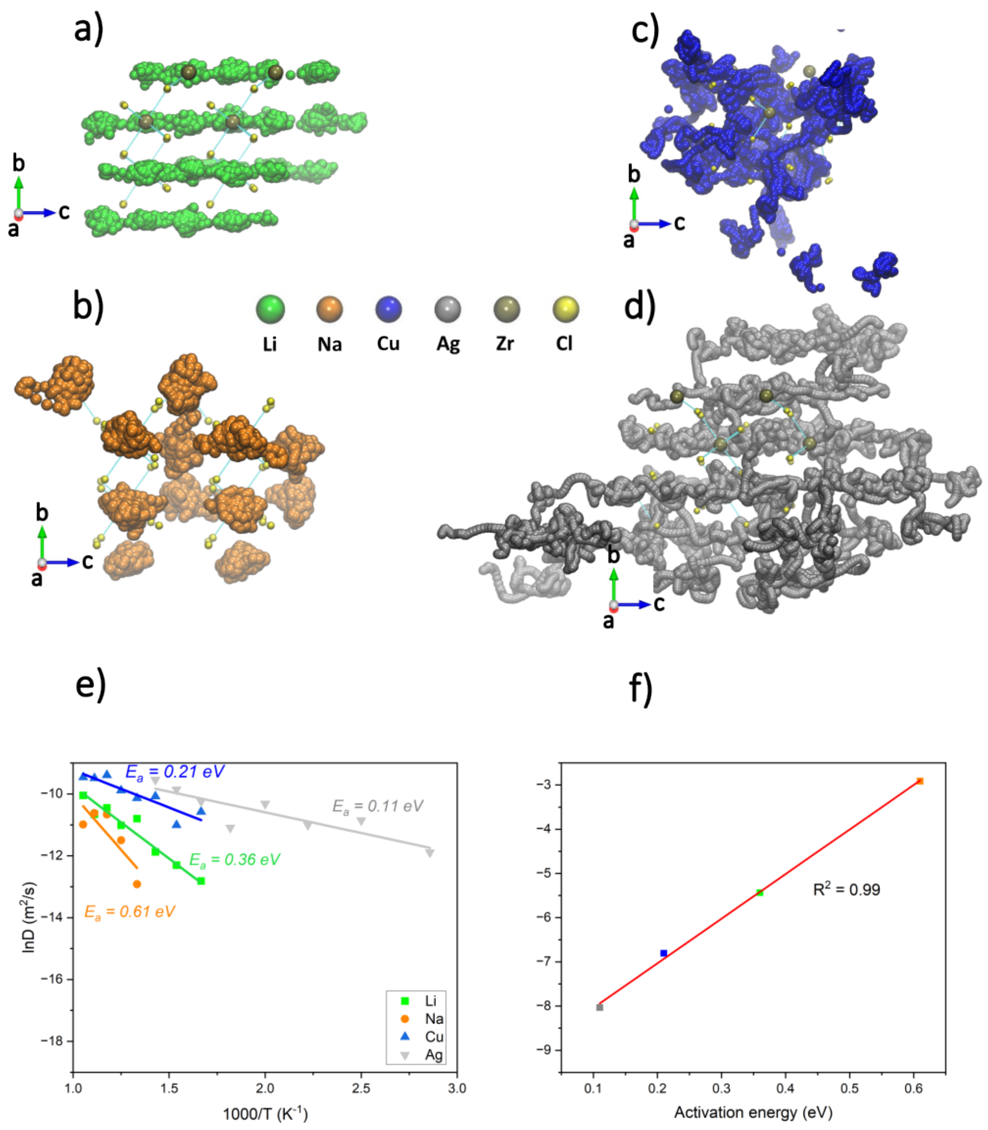

Supplementary Figure 6: Ab-initio molecular dynamics of $\boldsymbol{A_2ZrCl_6}$ materials. 50 ps AIMD trajectories at 600K of the mobile A^+ species in a) Li_2ZrCl_6, b) Na_2ZrCl_6, c) Cu_2ZrCl_6 and d) Ag_2ZrCl_6. Trajectories are smoothed over 50 frames. e) Arrhenius plots and the associated activation energies for ion diffusion in the different structures. f) Plot of activation energy vs pre-exponential factor demonstrating a Meyer-Neldel conductivity relationship.

Supplementary note 4: Molecular dynamics analysis

The ions exhibit vastly different mobility's at 600K. Li shows more displacement in the c-direction than in the ab-plane due to the lower energy c-axis barriers, as observed via transition state searching. Na shows a slight preference for diffusion in the c-direction, however the displacement is still smaller compared to Li. Cu and Ag are much more mobile in their respective structures. Ag again displays anisotropic diffusion favouring the c-direction, whilst Cu slightly favours transport in the ab-plane, which can be attributed to the connectivity of the tetrahedral sites in Cu_2ZrCl_6. The high mobility of Ag is interesting due to the structural similarities to the Li and Na analogues, despite Ag ions having larger ionic radii than both Li and Na when

octahedrally coordinated (1.15 Å vs 0.76 Å and 1.02 Å for Ag^+, Li^+ and Na^+ respectively[34]).

For the Na_2ZrCl_6 system, a discontinuity in the diffusion coefficient was observed below 700 K, which is consistent with previous molecular dynamics-based studies [27,37]. The activation energy for Na conduction was therefore extracted from a linear fit to the high temperature data points above 700K.

The discrepancy between our theoretical values and those experimentally observed may arise from the low crystallinity of our samples produced by mechanochemical synthesis. Obtaining single crystals of both Cu_2ZrCl_6 and Ag_2ZrCl_6 may provide further insight to the ionic mobility's of the A^+ ions.

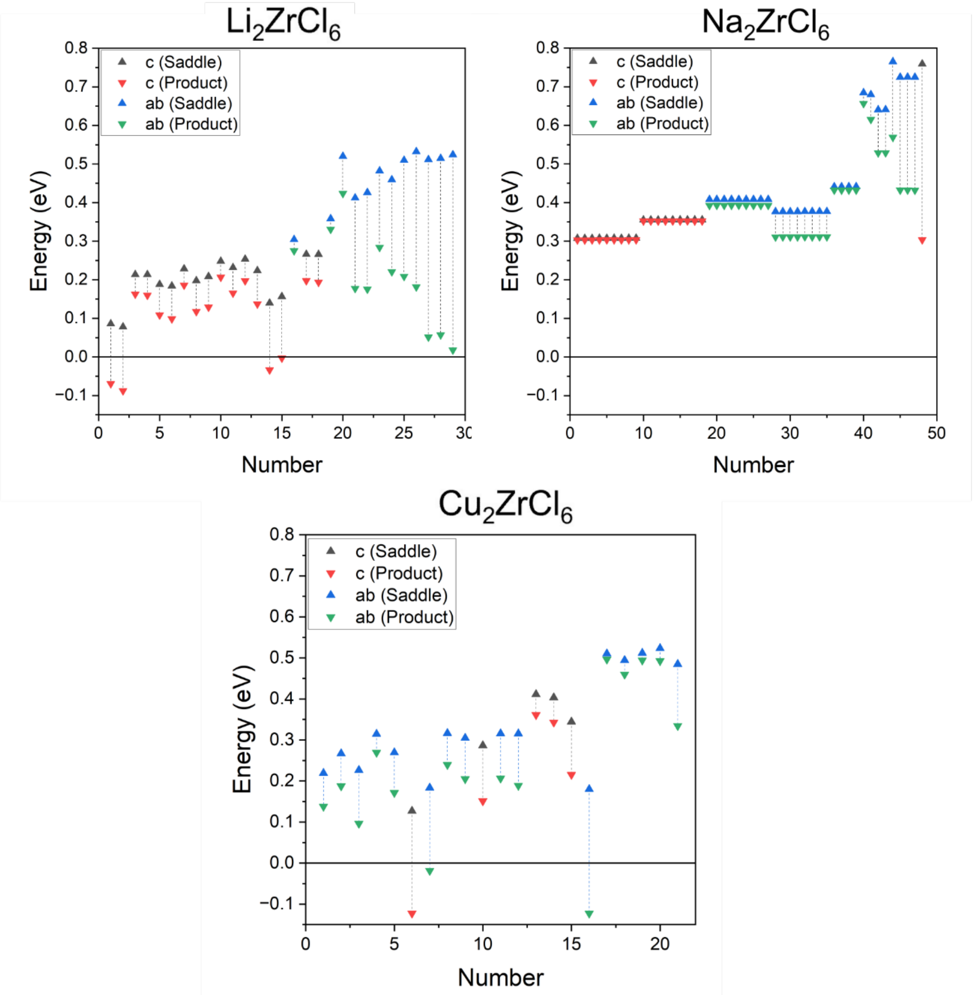

Supplementary Figure 7: Transition state searching calculations of ionic transport in *A*$_2$ZrCl$_6$ materials. Energies are relative to the reactant state i.e. the 'saddle' energy is calculated as: E_{Saddle} - $E_{Reactant}$.

Supplementary note 5: Analysis of transition state searching mechanisms

In Li_2ZrCl_6, any hop where the product state results in a new pair of face sharing $LiCl_6$-$LiCl_6$ is found to be higher than that of where the resulting product state is corner or edge sharing. No barriers were observed where a Li^+ ion moved to a position that is face sharing with Zr, suggesting that this process would have such a large barrier, it is unlikely to occur at room temperature.

The nature of the DFT ground state structure of Na_2ZrCl_6 means that any hop results in a new high energy face sharing pair (Na-Na or Na-Zr). This is directly observed by the barrier height for the forward and reverse directions; in the forward direction (product is face sharing) the activation energy is large while in the reverse (product is edge/corner sharing) the barrier is small < 0.1 eV. The forward or reverse barriers in Na_2ZrCl_6 also scale depending on whether it is transport in the ab-plane or along the c-channels (lower barriers in the c-direction). These observed anisotropic barriers suggest that the local coordination, such as Zr order/disorder, plays an important role in the available transport pathways within these materials.

No barriers were observed for Cu_2ZrCl_6 where the product state is face sharing with Zr, suggesting these sites are thermodynamically unfavourable. This leads to the bottleneck for diffusion in Cu_2ZrCl_6 being a tet-trig-oct-trig-tet hop to an adjacent cage.

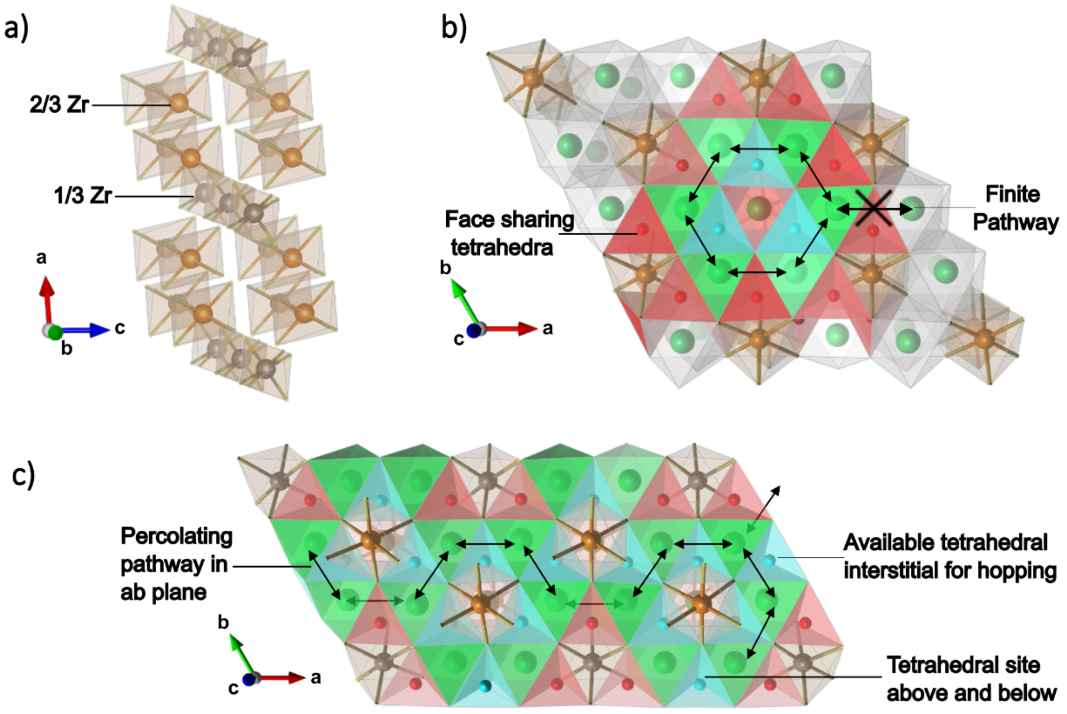

Supplementary Figure 8: Schematic representations of diffusion pathways in A_2ZrCl_6. a) An ordered zirconium sublattice in A_2ZrCl_6 showing different site occupation of Zr in the layers. At c = 0, Zr = 2/3 and at c = 0.5, Zr = 1/3. b) non-percolating (finite) pathway in a layer where 2/3 Zr sites are occupied. c) Percolating pathway in a layer where 1/3 Zr are occupied.

Supplementary note 6: Percolation theory analysis

Percolation theory, known for assessing long-distance diffusion via probability, relies on the concept of site percolation, which involves sites in a lattice that either 'open' or 'closed'. In the context of diffusion in A_2ZrCl_6, the sites are the A^+ positions. As shown by the TSS data, sites that are face-sharing with zirconium are thermodynamically inaccessible, thus considered as closed sites. While one-dimensional ion transport along the c-chains appears to require minimal energy due to the absence of these closed sites, the two-dimensional ab-plane presents a more intricate scenario. In this plane, the presence or absence of Zr dictate the diffusion properties, hence employing site percolation theory. This choice is justified by the impact of disorder across the zirconium sites in the layers of A_2ZrCl_6 on macroscopic diffusion behavior. Notably, the triangular/hexagonal 2D lattice formed by the zirconium sublattice in the ab-plane

exhibits a percolation threshold pc of 0.5, meaning that once the zirconium vacancy percentage p reaches or exceeds this value, a percolating pathway is guaranteed.

Supplementary Figure 8 depicts the network situation in different layers of A_2ZrCl_6 based on MD and TSS simulations. In one layer, where the Zr site occupancy is 2/3, the occupation probability falls below the percolation threshold ($p < pc$), resulting in the absence of a percolating network. The presence of only 6 accessible sites encircling a zirconium vacancy in the ab-plane restricts the A^+ ion's movement. Conversely, in another layer with a 1/3 zirconium site occupancy, the occupation probability surpasses the percolation threshold, enabling an infinitely percolating pathway across the lattice. This phenomenon is facilitated by the additional Zr vacancy in each unit cell, allowing A^+ ions to move freely around vacancies and between adjacent hexagons. The TSS data surprisingly indicates no significant difference in energies for diffusion in the c-direction across layers with differing Zr content. This finding implies that implementing percolation across all layers would result in uninhibited lithium ion diffusion.

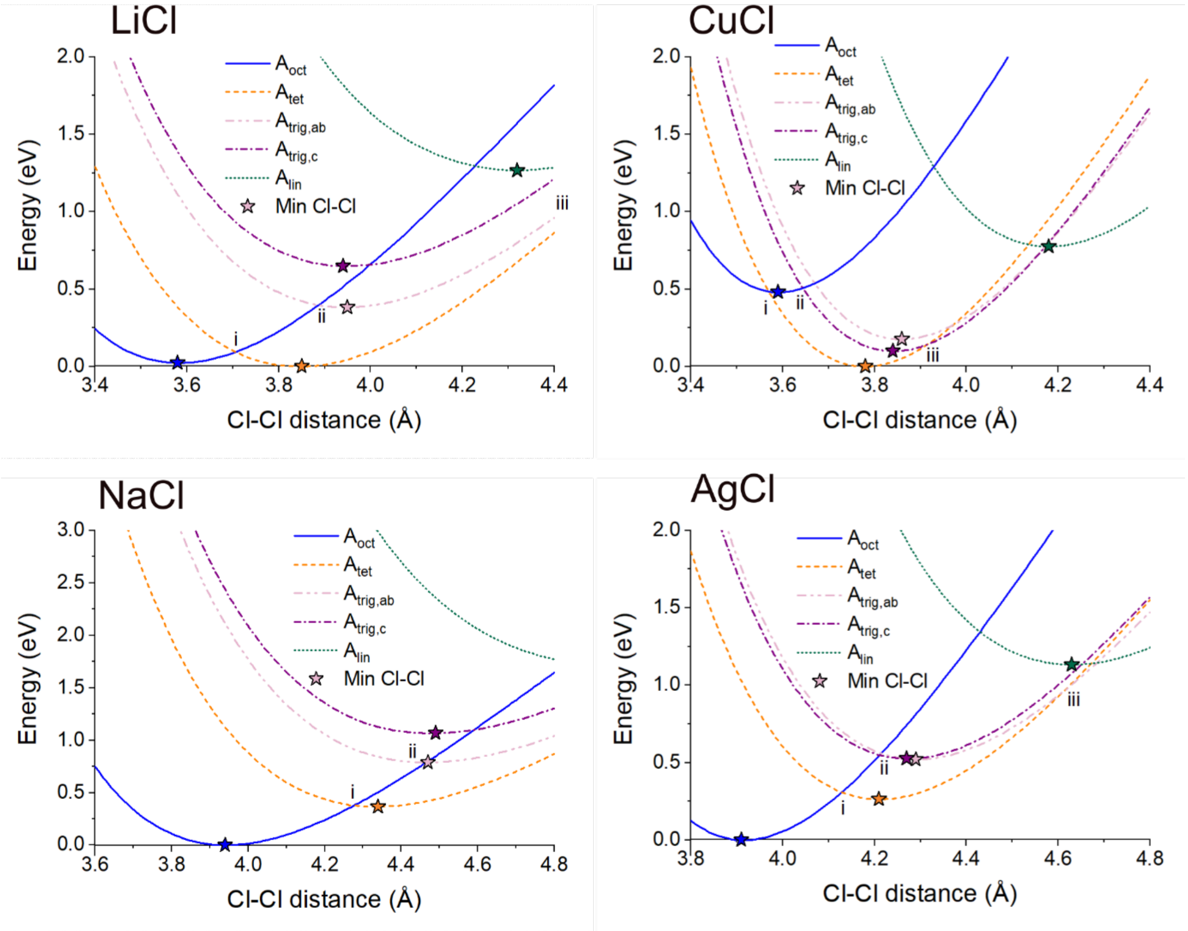

Supplementary Figure 9: Energy-volume relationships for HCP *A*Cl structures from DFT. Energies of different octahedral (A_{oct}), tetrahedral (A_{tet}), trigonal planar (A_{trig}) and linear (A_{lin}) A-site coordinations in model HCP ACl systems at varying Cl-Cl distances. Points i, ii and iii represent the distances at which the A_{oct}/A_{tet}, A_{oct}/A_{trig} and A_{tet}/A_{trig} energies are the same respectively. Stars indicate the minimum energy for each configuration.

Supplementary note 7: Site preference in model HCP *A*Cl systems

For HCP LiCl, the octahedral coordination shows a minimum at the smallest Cl-Cl bond distances (3.58 Å), whereas at longer bond distances (3.85 Å), the tetrahedral coordination becomes a minimum. The minimum energy tetrahedral Li configuration is predicted to be the ground state structure with r2SCAN calculations, with the octahedral configuration slightly higher in energy (24 meV/formula unit). The trigonal planar configurations along the ab ($A_{trig,ab}$) and c ($A_{trig,c}$) directions reaches a minimum in energy at X_{Cl-Cl} = 3.95 Å and 3.94 Å respectively. The trigonal planar configuration is higher in energy than the tetrahedral configuration for all Cl-Cl bond distances, and is thus expected to exist as a transition state configuration instead of a local minimum.

Similar behaviour is observed for the HCP NaCl and AgCl systems. For NaCl, A_{oct} is the ground state configuration (0 eV) when X_{Cl-Cl} = 3.94 Å. The minimum energy A_{tet}

(0.37 eV), $A_{trig,ab}$ (0.78 eV) and $A_{trig,c}$ (0.68 eV) configurations occur at Cl-Cl bond lengths of X_{Cl-Cl} = 4.43, 4.47 and 4.46 Å, respectively. For AgCl, A_{oct} is the ground state configuration when X_{Cl-Cl} = 3.91 Å, with minimum energy A_{tet} (0.26 eV), $A_{trig,ab}$ (0.52 eV) and $A_{trig,c}$ (0.49 eV) configurations at Cl-Cl bond lengths of X_{Cl-Cl} = 4.21, 4.29 and 4.30 Å, respectively.

The inverse behaviour is observed for the CuCl system. The tetrahedral Cu configuration shows a deep minimum at X_{Cl-Cl} = 3.78 Å. The octahedral configuration shows a minimum at X_{Cl-Cl} = 3.59 Å, but this configuration is 0.48 eV higher than the minimum A_{tet} configuration. This is consistent with the strong preference for tetrahedral coordination in Cu_2ZrCl_6. At bond lengths of X_{Cl-Cl} = 3.86 and 3.87 Å, the $A_{trig,ab}$ (0.18 eV) and $A_{trig,c}$ (0.20 eV) configurations, respectively, have minimum energies. The lower energy of the A_{trig} sites compared to the A_{oct} sites indicates that the A_{oct} site will be the transition state at long Cl-Cl bond distances.

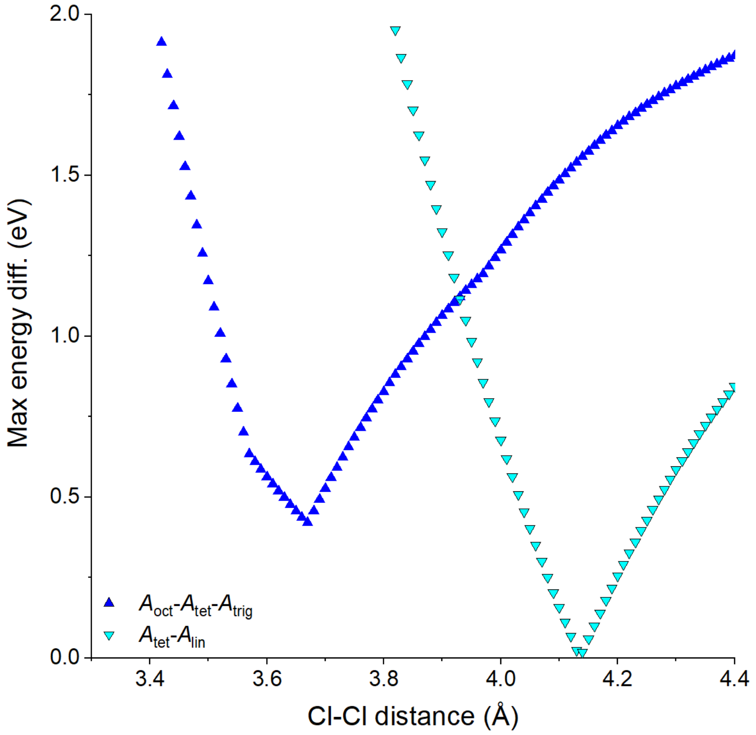

Supplementary Figure 10: DFT energy volume relationship for CuCl. Plot of maximum energy difference per formula unit between A_{oct} - A_{tet} - $A_{trig,ab}$ sites (blue) and A_{tet} – A_{lin} sites (cyan) in HCP CuCl.

Supplementary note 8: Role of linear configurations in HCP CuCl.

In Supplementary Figure 9, the energy of linear (2 coordinate) Cu configurations was also investigated as Cu_{tet}-Cu_{lin}-Cu_{tet} pathways were observed in Cu_2ZrCl_6 through transition state searching. Supplementary Figure 10 shows that when the Cl-Cl distance in HCP CuCl becomes longer than $X_{cl\text{-}cl}$ = 3.93 Å, the A_{tet} – A_{lin} - A_{tet} pathway becomes increasingly lower in energy than the previous A_{tet} – A_{trig} – A_{oct} – A_{trig} - A_{tet} pathway. Importantly, there is only a single change in coordination for the $A_{tet}(4)$ – $A_{lin}(2)$ - $A_{tet}(4)$ pathway; there is a single point at which the two configurations have the same energy ($X_{Cl\text{-}Cl}$ = 4.14 Å), leading to zero intrinsic barrier, analogous to the A_{oct} - $A_{trig,c}$ pathways in Figure 4e. The A_{lin} pathways were also calculated for LiCl, NaCl and AgCl, but the A_{tet} – A_{lin} - A_{tet} pathway was found to be higher in energy than the A_{tet} – A_{trig} – A_{oct} – A_{trig} - A_{tet} pathways until very long $X_{Cl\text{-}Cl}$ distances, making this pathway inaccessible.

Supplementary Table 1. Rietveld refinement result from the room-temperature X-ray powder diffraction data of the as-milled Li_2ZrCl_6. Uncertainties are in brackets, fixed parameters are shown with (-). The space group is $P\bar{3}m1$. The refined lattice parameters are a = 10.968(4) Å and c = 5.931(21) Å.

**Atoms**	**x**	**y**	**z**	**Occ.**	**site**	**Sym.**	**U_{iso}**
Li1	0.316(7)	0	0	0.88	6g	1	0.091(9)
Li2	0.330(11)	0	½	0.12	6h	1	0.073
Zr1	0	0	0	0.73	1a	2	0.083(5)
Zr2	1/3	2/3	0.485(15)	0.36	2c	2	0.040(29)
Zr3	0	0	½	0.43	1b	6	0.012(-)
Zr4	1/3	2/3	0.015(11)	0.48	2d	6	0.010(24)
Cl1	0.107(5)	0.892	0.770(9)	1	6i	6	0.035(21)
Cl2	0.224(4)	0.775	0.263(7)	1	6i	6	0.044(24)
Cl3	0.439(4)	0.561	0.779(8)	1	6i	6	0.044(28)

Supplementary Table 2. Rietveld refinement result from the room-temperature X-ray powder diffraction data of the as-milled Na_2ZrCl_6. Uncertainties are in brackets, fixed parameters are shown with (-). The space group is $P\bar{3}m1$. The refined lattice parameters are a = 11.491(12) Å and c = 6.263(7) Å.

**Atoms**	**x**	**y**	**z**	**Occ.**	**site**	**Sym.**	**U_{iso}**
Zr1	0	0	0	0.70	1a	1	0.006(3)
Zr2	1/3	2/3	0.543(9)	0.75	2d	1	0.007(26)
Zr3	0	0	½	0.55	1b	2	0.029(4)
Na1	0.353(22)	0	0	0.50	6g	6	0.088(7)
Na2	0.339(23)	0	½	0.50	6h	6	0.069(7)
Cl1	0.108(6)	0.892	0.222(13)	1	6i	6	0.066(5)
Cl2	0.232(7)	0.768	0.758(14)	1	6i	6	0.066(4)
Cl3	0.429(5)	0.571	0.230(15)	1	6i	6	0.148(6)

Supplementary Table 3. Rietveld refinement result from the room-temperature X-ray powder diffraction data of the as-milled Cu_2ZrCl_6. Uncertainties are in brackets, fixed parameters are shown with (-). The space group is $P\bar{3}m1$. The refined lattice parameters are a = 10.987(9) Å and c = 6.132(32) Å.

Atoms	x	y	z	Occ.	site	Sym.	U_{iso}
Zr1	1/3	2/3	0.987(23)	0.52	2c	2	0.002(-)
Zr2	0	0	½	0.51	1b	1	0.032(4)
Zr3	1/3	2/3	0.540(26)	0.29	2d	2	0.010(5)
Zr4	0	0	0	0.67	1a	2	0.067(5)
Cu1	0.231(6)	0.769	0.440(17)	0.40	6i	6	0.073(5)
Cu2	0.110(9)	0.890	0.900(31)	0.21	6i	6	0.020(-)
Cu3	0.460(4)	0.540	0.575(19)	0.030	6i	6	0.041(-)
Cu4	0.218(4)	0.782	0.089(14)	0.060	6i	6	0.050(-)
Cu5	0.893(6)	0.108	0.406(22)	0.31	6i	6	0.042(4)
Cl1	0.441(6)	0.559	0.208(16)	1	6i	6	0.036(4)
Cl2	0.103(5)	0.897	0.268(12)	1	6i	6	0.025(4)
Cl3	0.227(7)	0.773	0.764(20)	1	6i	6	0.032(6)

Supplementary Table 4. Rietveld refinement result from the room-temperature X-ray powder diffraction data of the as-milled Ag_2ZrCl_6. Uncertainties are in brackets, fixed parameters are shown with (-). The space group is $P\bar{3}m1$. The refined lattice parameters are a = 11.383(11) Å and c = 6.138(5) Å.

Atoms	x	y	z	Occ.	site	Sym.	U_{iso}
Zr1	0	0	½	0.75	1b	1	0.065(-)
Zr2	0	0	0	0.30	1a	1	0.089(-)
Zr3	1/3	2/3	0.505(15)	0.85	2d	2	0.061(25)
Zr4	1/3	2/3	0.091(10)	0.10	2d	2	0.003(-)
Ag1	0.336(5)	0	0	0.86	6g	6	0.058(16)
Ag2	0.312(9)	0	½	0.14	6h	6	0.042(-)
Cl1	0.201(5)	0.799	0.264(14)	1	6i	6	0.006(4)
Cl2	0.127(6)	0.873	0.823(16)	1	6i	6	0.048(6)
Cl3	0.559(6)	0.441	0.265(20)	1	6i	6	0.025(6)

Supplementary Table 5. Rietveld refinement result from the room-temperature X-ray powder diffraction data of the as-milled Na_2ZrCl_6. Uncertainties are in brackets, fixed parameters are shown with (-). The space group is $P2_1/n$. The refined lattice parameters are a = 6.674(9) Å, b = 7.083(10) Å c = 9.817(13) Å. β = 92.224°.

Atoms	x	y	z	Occ.	site	Sym.	U_{iso}
Zr1	½	½	½	1	2b	2	0.036(4)
Na1	0.530(4)	0.085(3)	0.287(24)	1	4e	4	0.021(-)
Cl1	0.266(27)	0.203(31)	0.941(20)	1	4e	4	0.059(11)
Cl2	0.107(26)	0.95(4)	0.238(21)	1	4e	4	0.073(9)
Cl3	0.652(27)	0.773(25)	0.440(19)	1	4e	4	0.013(8)

Supplementary references

1. Asano, T. *et al.* Solid halide electrolytes with high lithium-ion conductivity for application in 4 V class bulk-type all-solid-state batteries. *Advanced Materials* **30,** (2018).
2. Peng, H., Machida, N. & Shigematsu, T. Mechano-chemical synthesis of $RbAg_4I_5$ and KAg_4I_5 crystals and their silver ion conducting properties. *Journal of the Japan Society of Powder and Powder Metallurgy* **49,** 69–74 (2002).
3. Abudouwufu, T. *et al.* Crystal structure and copper ion emission properties of $Rb_4Cu_{16}I_7Cl_{13}$ solid electrolyte. *Vacuum* **196,** 110742 (2022).
4. Li, X. *et al.* Water-mediated synthesis of a superionic halide solid electrolyte. *Angewandte Chemie* **131,** 16579–16584 (2019).
5. Liang, J. *et al.* Site-occupation-tuned superionic Li_xScCl_{3+x} halide solid electrolytes for all-solid-state batteries. *Journal of the American Chemical Society* **142,** 7012–7022 (2020).
6. Schlem, R. *et al.* Mechanochemical synthesis: A tool to tune cation site disorder and ionic transport properties of Li_3MCl_6 (m = Y, Er) superionic conductors. *Advanced Energy Materials* **10,** (2019).
7. Kim, S. Y. *et al.* Lithium ytterbium-based halide solid electrolytes for high voltage all-solid-state batteries. *ACS Materials Letters* **3,** 930–938 (2021).
8. Kwak, H. *et al.* All-solid-state batteries: New cost-effective halide solid electrolytes for all-solid-state batteries: Mechanochemically prepared Fe^{3+} substituted Li_2ZrCl_6. *Advanced Energy Materials* **11,** (2021).
9. Kwak, H. *et al.* Na_2ZrCl_6 enabling highly stable 3V all-solid-state Na-Ion batteries. *Energy Storage Materials* **37,** 47–54 (2021).
10. Court-Castagnet, R. Ionic conductivity-enhancement of LiCl by homogeneous and heterogeneous dopings. *Solid State Ionics* **61,** 327–334 (1993).
11. Kanno, R., Takeda, Y., Masuyama, Y., Yamamoto, O. & Takahashi, T. Phase diagram and high copper ion conductivity of the copper(I) chloride-rubidium chloride system. *Solid State Ionics* **11,** 221–226 (1983).
12. Shao, C. *et al.* Structure and ionic conductivity of cubic $Li_7La_3Zr_2O_{12}$ solid electrolyte prepared by chemical co-precipitation method. *Solid State Ionics* **287,** 13–16 (2016).
13. Zhao, Y. & Daemen, L. L. Superionic conductivity in lithium-rich Anti-Perovskites. *Journal of the American Chemical Society* **134,** 15042–15047 (2012).

14. Kamaya, N. *et al.* A lithium superionic conductor. *Nature Materials* **10,** 682–686 (2011).
15. Briant, J. L. & Farrington, G. C. Ionic conductivity in Na^+, K^+, and Ag^+ β-alumina. *Journal of Solid State Chemistry* **33,** 385–390 (1980).
16. Zhang, Z. *et al.* $Na_3Zr_2Si_2PO_{12}$: A stable Na^+ ion solid electrolyte for solid-state batteries. *ACS Applied Energy Materials* **3,** 7427–7437 (2020).
17. Inaguma, Y. *et al.* High ionic conductivity in lithium lanthanum titanate. *Solid State Communications* **86,** 689–693 (1993).
18. Bernuy-Lopez, C. *et al.* Atmosphere controlled processing of Ga-substituted garnets for high Li-ion conductivity ceramics. *Chemistry of Materials* **26,** 3610–3617 (2014).
19. Le Van-Jodin, L., Ducroquet, F., Sabary, F. & Chevalier, I. Dielectric properties, conductivity and Li^+ ion motion in LIPON thin films. *Solid State Ionics* **253,** 151–156 (2013).
20. Li, W. *et al.* Li^+ ion conductivity and diffusion mechanism in α-LI_3N and β-LI_3N. *Energy & Environmental Science* **3,** 1524 (2010).
21. Pogodin, A. I., Filep, M. J., Izai, V. Yu., Kokhan, O. P. & Kúš, P. Crystal growth and electrical conductivity of Ag_7PS_6 and Ag_8GeS_6 Argyrodites. *Journal of Physics and Chemistry of Solids* **168,** 110828 (2022).
22. Kuhs, W. F., Nitsche, R. & Scheunemann, K. The argyrodites — a new family of tetrahedrally close-packed structures. *Materials Research Bulletin* **14,** 241–248 (1979).
23. Hayashi, A., Noi, K., Sakuda, A. & Tatsumisago, M. Superionic glass-ceramic electrolytes for room-temperature rechargeable sodium batteries. *Nature Communications* **3,** (2012).
24. Wang, S. *et al.* High-conductivity argyrodite Li_6PS_5Cl solid electrolytes prepared via optimized sintering processes for all-solid-state lithium–sulfur batteries. *ACS Applied Materials & Interfaces* **10,** 42279–42285 (2018).
25. Seino, Y., Ota, T., Takada, K., Hayashi, A. & Tatsumisago, M. A aulphide lithium super ion conductor is superior to liquid ion conductors for use in rechargeable batteries. *Energy Environ. Sci.* **7,** 627–631 (2014).
26. Bron, P. *et al.* $Li_{10}SiP_2S_{12}$: An affordable lithium superionic conductor. *Journal of the American Chemical Society* **135,** 15694–15697 (2013).
27. Sebti, E. *et al.* Synthetic control of structure and conduction properties in Na–Y–Zr–Cl solid electrolytes. *Journal of Materials Chemistry A* **10,** 21565–21578 (2022).
28. Dattelbaum, A. M., He, L., Tsui, F. & Martin, J. D. Synthesis and characterization of Cu_2ZrCl_6: A thermochromic, Van Vleck paramagnet. *Journal of Alloys and Compounds* **338,** 173–184 (2002).
29. Takahashi, T. Solid silver ion conductors. *Journal of Applied Electrochemistry* **3,** 79–90 (1973).
30. Suzuki, M. & Okazaki, H. The structure of α-AgI. *Physica Status Solidi (a)* **42,** 133–140 (1977).
31. Schlem, R., Banik, A., Ohni, S., Suard, E. & Zeier, W. Insights into the lithium substructure of the superionic conductors Li_3YCl_6 and Li_3YBr_6. *Chemistry of Materials*, **33**, 327-337 (2021).
32. Bohnsack, A. *et al.* Ternäre halogenide vom typ A_3MX_6. VI. Ternäre Chloride der selten-erd-elemente mit lithium, Li_3MCl_6 (M = Tb-Lu, Y, Sc): Synthese, Kristallstrukturen und Ionenbewegung. *Zeitschrift für anorganische und allgemeine Chemie* **623,** 1067–1073 (1997).

33. Schmidt, M. O., Wickleder, M. S. & Meyer, G. Zur Kristallstruktur von Li_3InCl_6. *Zeitschrift für anorganische und allgemeine Chemie* **625,** 539–540 (1999).
34. Shannon, R. D. Revised effective Ionic radii and systematic studies of interatomic distances in halides and chalcogenides. *Acta Crystallographica Section A* **32,** 751–767 (1976).
35. Paul, R. & Thangadurai, V. Understanding transport properties of conducting solids: Meyer-Neldel Rule Revisited. *Ionics* **27,** 4917–4925 (2021).
36. Henkelman, G., Arnaldsson, A. & Jónsson, H. A fast and robust algorithm for Bader decomposition of charge density. Computational Materials Science **36**, 354–360 (2006).
37. Wu, E. et al. A stable cathode-solid electrolyte composite for long-cycle-life, high voltage solid-state sodium-ion batteries. Nature Communications **12** 1256 (2020).
